# A Prospective Comparison of Standard and Modified Acute Malnutrition Treatment Protocols during COVID-19 in South Sudan

**DOI:** 10.3390/nu15234853

**Published:** 2023-11-21

**Authors:** Shannon Doocy, Sarah King, Sule Ismail, Eva Leidman, Heather Stobaugh, The Action Against Hunger/US Centers for Disease Control and Prevention Study Team

**Affiliations:** 1 Johns Hopkins Bloomberg School of Public Health, Johns Hopkins University, Baltimore, MD 21205, USA; 2 Action Against Hunger, New York, NY 10004, USA; unl6@cdc.gov (S.K.); hstobaugh@actionagainsthunger.org (H.S.); 3 US Centers for Disease Control and Prevention, Atlanta, GA 30333, USA; ismail@integralglobal.net (S.I.); wzu0@cdc.gov (E.L.); 4 Integral Global Consulting, Atlanta, GA 30084, USA

**Keywords:** simplified nutrition treatment protocol, COVID-19 nutrition adaptations, acute malnutrition, community management of acute malnutrition, South Sudan

## Abstract

A non-randomized prospective cohort study was conducted in 2022 to compare recovery rate and length of stay (LoS) for acutely malnourished children treated under South Sudan’s standard Community Management of Acute Malnutrition (CMAM) protocol and a COVID-modified protocol. Children aged 6–59 months received acute malnutrition (AM) treatment under the standard or modified protocol (mid-upper-arm circumference-only entry/exit criteria and simplified dosing). Primary (recovery rate and LoS) were compared for outpatient therapeutic (OTP) and therapeutic supplementary feeding programs (TSFP) using descriptive statistics and mixed-effects models. Children admitted to OTP under both protocols were similar in age and sex; children admitted to TSFP were significantly older under the modified protocol than the standard protocol. Shorter LoS and higher recovery rates were observed under the modified protocol for both OTP (recovery: 93.3% vs. 87.2%; LoS: 38.3 vs. 42.8 days) and TSFP (recovery: 79.8% vs. 72.7%; LoS: 54.0 vs. 61.9 days). After adjusting for site and child characteristics, neither differences in adjusted odds of recovery [OTP: 2.63; TSFP 1.80] nor LoS [OTP −10.0; TSFP −7.8] remained significant. Modified protocols for AM performed well. Adjusted models indicate similar treatment outcomes to the standard protocol. Adopting simplified protocols could be beneficial post-pandemic; however, recovery and relapse will need to be monitored.

## 1. Introduction

Acute malnutrition affects 45.4 million children under five each year, placing them at increased risk for morbidity and mortality [1,2,3]. The COVID-19 pandemic exacerbated the situation, placing an additional 9.3 million children at risk; additionally, the need to mitigate COVID-19-transmission risk resulted in widespread modifications to treatment protocols, where simplified approaches were adopted rapidly at scale [4,5]. The majority of children with Severe and Moderate Acute Malnutrition (SAM and MAM) are treated on an outpatient basis in Community Management of Acute Malnutrition (CMAM) programs. Simplified approaches to CMAM include a range of adaptations (e.g., use of a single therapeutic product to treat SAM and MAM, simplified dosing, reduced visit frequency, admission based only on Mid-Upper Arm Circumference (MUAC)) intended to improve coverage and reduce cost [6]. Different combinations of simplified approaches enable the optimization of CMAM programs based on context and health service approaches, and the use of simplified approaches is increasingly common and accepted.

A substantial evidence base exists, and recent literature reviews indicate that simplified approaches can increase the number of children identified and treated for wasting; changes to dosing can improve the efficiency of Ready-To-Use Therapeutic and Supplementary Foods (RUTF/RUSF) use, while maintaining a high quality of care [6,7]. Use of simplified approaches was determined to be appropriate in exceptional circumstances by UN agencies in 2019 [8,9]. In South Sudan, simplified treatment protocols were adopted in various formats nationally in early 2020; Action Against Hunger, a global humanitarian organization, was given exceptional permission by the Ministry of Health to continue providing treatment using standard treatment protocols at sites enrolling for an ongoing study on acute-malnutrition relapse. Leveraging this opportunity, a non-randomized prospective cohort study was conducted in 2022 to compare outcomes of a standard CMAM protocol (implemented at Relapse Study Sites) to a COVID-modified protocol in terms of standard nutrition program indicators (recovery rate, Length of Stay (LoS)) [10].

## 2. Materials and Methods

Outcomes of acutely malnourished children aged 6–59 months treated under two different protocols, South Sudan’s standard CMAM protocol, and a COVID-modified protocol, were compared in a non-randomized prospective cohort study (Table 1) [11,12]. The COVID-modified protocol included MUAC-only admissions and discharges (whereby MUAC is the sole criterion for admitting and discharging children from treatment) with simplified dosing of RUSF in the management of MAM. Seven facilities in Aweil East County, Northern Bahr el Ghazal participated in the study, including three that implemented the standard protocol and four that used the modified protocol. Children were enrolled in the study at admission into either the Outpatient Therapeutic Program (OTP, for SAM) or Therapeutic Supplementary Feeding (TSFP, for MAM) and followed through program exit. The primary outcome measures were standard CMAM program indicators including LoS in the treatment program defined as days between admission and discharge and recovery rate defined as the proportion of children discharged as recovered.

Sample size was calculated to achieve reasonable power for estimating differences in mean LoS. Minimum sample size calculations were conducted in STATA 15 (College Station, TX) based on the following assumptions: (1) power (1-β) of 80% and significance of α = 0.05; (2) mean difference in LoS of 14 days with a standard deviation of 30 days based on program data); (3) two-sided comparison; (4) design effect of 1.5 to account for clustering in sites; and (5) a 10% loss to follow-up rate. The minimum required sample size per group was 123, which translates to a minimum sample of 246 children per treatment program. Sample size was doubled, to 1000 cases (1:1 ratio of OTP to TSFP), to allow for this power in sub-analysis among younger and older children.

Data collection occurred between February and November 2022. All children between 6–59 months diagnosed with SAM without medical complications or MAM presenting for treatment were eligible to participate; differences in enrollment criteria according to the protocol are presented in Table 1. Children were excluded if they presented with medical complications, were referred for inpatient treatment, were outside the stated age range, or if their caregiver did not consent. Caregivers were read a consent script and asked for verbal consent to participate in the study when their child was admitted to OTP or TSFP. Caregivers were invited to enroll in the study at the same visit the child was enrolled in OTP or TSFP; in instances where no study data collector was present, enrollment interviews were conducted after the initial visit. Efforts were made to recruit at least 250 children in OTP and TSFP per protocol, and enrollment of children with SAM took longer than children with MAM because OTP caseloads were smaller. The proportion of children enrolled in OTP and TSFP that were offered the opportunity to participate in the study differed by facility and study group because of the stratified sampling approach and differences in caseload size between facilities. Recruitment was not always consecutive and also depended on interviewer presence at the CMAM sites, with efforts made to contact caregivers of children enrolled in OTP/TSFP on days when study interviewers were not present.

A tablet-based enrollment questionnaire was administered via the Open Data Kit (ODK) platform at admission. The survey captured individual (child and caregiver) and household sociodemographic and economic characteristics, including food security. Surveys were written in English and verbally translated into Dinka and administered by data collectors from the study area with prior data collection experience following three-day training. Treatment data including dates of admission and discharge (to calculate LoS), sex, age in months, anthropometry (weight, height, and MUAC) at enrollment and discharge, and discharge outcome were extracted from program records collected at admission and discharge. All surveys and records were checked for completeness and uploaded weekly to a Microsoft Access database on a secure server. Real-time monitoring and double entry were conducted to ensure the consistency of the data, with follow-up verification as needed.

Variables were reviewed for completeness, consistency, and plausibility. Children were excluded from analysis if age at admission fell outside of 6–59 months, anthropometrics were extreme (outside ±5 or 6 standard deviations for various anthropometric parameters), or LoS was >120 days in either the OTP or TSFP component of treatment. Descriptive analysis with t-tests and chi-square tests were run on the corresponding binary, categorical, and continuous variables. Mixed models were used to assess the association of CMAM protocol with recovery and LoS outcomes. Odds of recovery were estimated with logistic regression, and differences in average LoS with linear regression models. Models included site-level random intercepts to adjust for the potential clustering of observations within sites and to adjust for the site-based protocol assignment. Individual and household-level characteristics at enrollment were included in the models as fixed effects. For the TSFP analysis, data included both children that transferred after exiting OTP and children starting treatment in TSFP. Except for age and MUAC at the time of TSFP transfer, covariates for transfer children are taken from the start of OTP; TSFP models also include an indication of if the child was a transfer or direct admission. Bayesian mixed models were fit using *rstanarm* version 2.21.3 with default priors [13,14,15] and 95% credible intervals, which represent the central portion of the range containing 95% of values in the distribution, are presented. All analyses were performed in R 4.0.3 (R Core Team 2020 [16]).

The study was approved by the South Sudan Ministry of Health Ethics Committee and the Johns Hopkins Bloomberg School of Public Health Institutional Review Board. This activity was reviewed by the US Center for Disease Control and Prevention (CDC) and was conducted in a manner consistent with federal law and CDC policy.

## 3. Results

**Figure 1:**
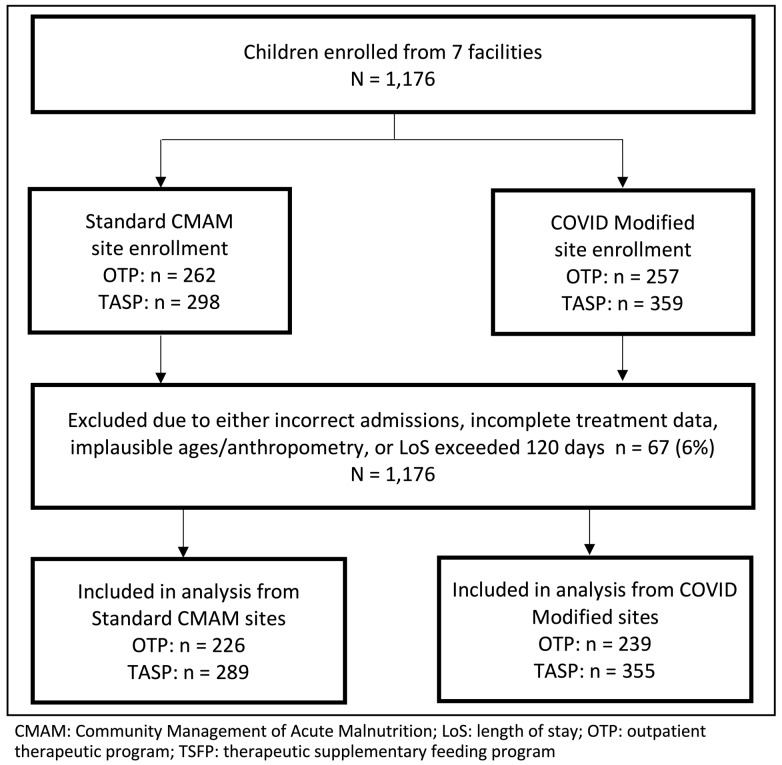
Participant flow diagram.

The study enrolled 1176 children, of which 1109 (94.3%) were included in the final analyses (Figure 1). There were significant differences between children enrolled in the two study arms (Table 2), which are likely due to the standard-protocol sites being in larger population centers and located on major thoroughfares. Children with SAM that enrolled in OTP were similar in terms of age, sex, and breastfeeding status; children with MAM that enrolled in TSFP at standard CMAM sites were significantly younger and more likely to be breastfed compared to modified-protocol sites. Compared to standard-protocol sites, households of children enrolled at modified-protocol sites were significantly larger; more food insecure and less likely to receive food assistance; less likely to be displaced; more likely to have married caregivers; and resided slightly closer to treatment facilities. Differences are likely related to modified-protocol sites being more rural with lesser access to markets, transportation, and ongoing services. Among children with SAM admitted to OTP, MUAC at admission was significantly higher under the standard protocol than under the modified protocol (11.5 cm vs. 11.2 cm, *p* < 0.001); differences in WHZ on admission could not be evaluated given weight and height were only evaluated in sites implementing the standard protocol. MUAC of children admitted to TSFP for MAM treatment under the two protocols was similar (*p* = 0.13) (Table 3).

There were differences in nutritional status at exit and treatment outcomes between the two protocols. Among children exiting OTP, mean MUAC remained significantly higher at exit among children treated under the standard protocol (12.0 cm vs. 11.8 cm; *p* < 0.001). However, the mean MUAC gain was slightly greater under the modified protocol (0.6 cm vs. 0.5 cm, *p* = 0.02). These differences were not observed with TSFP treatment, where both MUAC at exit and MUAC gain were similar under the two protocols. Average daily MUAC gain was, however, significantly greater under the modified protocol for both OTP and TSFP (*p* ≤ 0.002 for both comparisons). 

Compared to the standard protocol, LoS was significantly shorter under the modified protocol for OTP and TSFP, respectively (OTP: 42.8 vs. 38.3 days; *p* = 0.03; TSFP (61.9 vs. 54.0 days, *p* < 0.001). Among recovered children, time to recovery was similar under the two protocols for OTP (40.2 days standard vs. 37.0 days modified, *p* = 0.12) and significantly shorter under the modified protocol for TSFP (61.3 days standard vs. 51.2 days modified, *p* < 0.001). In comparing the recovery rates between the standard and adapted protocols, the modified protocol had significantly higher recovery rates for both OTP (87.2% standard vs. 93.3% modified, *p* = 0.03) and TSFP (72.7% standard vs. 79.8% modified, *p* = 0.01). More children were non-responsive (i.e., not recovering in the expected timeframe) under the standard protocol in both OTP and TSFP, and under the standard protocol in TSFP, transfers and improper discharge were more frequent.

Mixed-effect models, adjusting for enrollment site and measured differences in child characteristics on admission, showed that the modified protocol was associated with an average decrease in LoS of 10.0 days (Credibility Interval (CrI): −25.3, 4.7) in OTP and 7.8 days (CrI: −21.7, 6.7) in TSFP (Table 4); findings were similar when children admitted on low WHZ were excluded from the analysis (Appendix A). Credibility intervals of 95% for both OTP and TSFP overlap zero, suggesting that after adjusting for enrollment site and measured differences in child characteristics on admission differences in length of stay were not significant. MUAC at admission was associated with LoS in both OTP and TSFP. For each mm increase in MUAC on admission, LoS decreased by 1.1 days (CrI: −1.5, −0.6) in OTP and 1.3 days (CrI: −1.8, −0.8) in TSFP. The child’s sex and age at admission were not associated with LoS.

Children treated under the modified protocol had higher odds of recovery compared to the standard protocol in both OTP (adjusted odds ratio (AOR) = 2.63, CrI: 0.70, 9.09) and TSFP (AOR = 1.80, CrI: 0.42, 7.24). As with LoS, 95% credibility intervals for both OTP and TSFP overlap by one, suggesting that after adjustment differences in recovery rates were not significant. When adjusted models were run without children admitted on low WHZ, the odds of recovery remained similar (Appendix A). Child age and sex were not associated with recovery in adjusted models; however, MUAC at admission was associated with increases of 16% and 28% in odds of recovery in OTP and TSFP, respectively (OTP AOR = 1.16, CrI: 1.08, 1.25; TSFP AOR = 1.28, CrI: 1.19, 1.38).

While recovery rates for OTP and TSFP were satisfactory, concerns about continuity of care emerge (under both protocols) when full recovery of SAM children is considered, which is defined as a child with SAM recovering fully from acute malnutrition (i.e., progressing from SAM to MAM to not malnourished). Of all children admitted to OTP in the study (i.e., combined analysis under both protocols), 39% failed to transfer to TSFP (i.e., children treated for SAM that never enroll in treatment for MAM) which is surprising given that treatment for both is provided at the same facilities. This translates to a 43.5% default rate and a SAM-recovery rate of only 34.4%, which is well below the 75% standard [17]. Compared to children admitted to TSFP directly, OTP transfers had worse TSFP outcomes, including lower recovery rates (56.6% vs. 84.3%, *p* < 0.001) and higher likelihood of transfer back to OTP (13.7% vs. 5.4%); the average LoS was also significantly longer among OTP transfers (65.6 vs. 54.6 days, *p* < 0.001). 

## 4. Discussion

Children treated under the modified protocol in OTP and TSFP had significantly shorter LoS and higher recovery rates. In both OTP and TSFP, average daily MUAC gain was significantly higher under the modified protocol. However, we cannot attribute these differences entirely to the treatment program given meaningful differences in child and household characteristics across study arms. Children admitted to OTP and TSFP under the modified protocol were significantly older than those admitted under the standard protocol, but there was no difference in sex composition; mean MUAC at admission was significantly higher among children admitted under the standard protocol in OTP only. Accounting for site of enrollment and child characteristics on admission, adjusted models showed increased odds of recovery and shorter LoS under the modified protocol, but results were not statistically significant. Mean MUAC was significantly associated with recovery and LoS but not age or sex in adjusted models. Findings from this study align with other recent evidence indicating that modified protocols were associated with improved CMAM outcomes during COVID-19 in South Sudan. A national analysis of CMAM program trends from 2019 to 2021 observed that OTP and TSFP recovery rates increased by 3.7% and 2.8%, respectively, during COVID-19 [18]. Another recent analysis of children treated for OTP in five states of South Sudan observed significantly higher recovery rates and odds of recovery under modified protocols compared to the standard protocol (AORs: 1.8–2.4, depending on the protocol) and significantly shorter LoS among all modified protocols [19].

MUAC-only admissions and discharge criteria are the most common CMAM protocol simplifications, both prior to and during the COVID-19 pandemic [6,20,21,22,23,24,25]. However, MUAC and WHZ measurements do not always identify the same children as acutely malnourished, such that differences in recovery and length of stay may be primarily driven by differences in patient profile resulting from the admission criteria [22,23,24]. While several studies have shown MUAC to be more sensitive than WHZ for mortality identification, more recent re-analysis has shown that children with both low MUAC and low WHZ are at greater risk of mortality [21,22,23,26]. In this study, 59% and 90% of children admitted to OTP and TSFP, respectively, qualified based on MUAC criteria. Another study in the same area of Northern Bahr el Gazal, where the predominate ethnic group (Dinka) are typically tall and slim, observed that 67% of OTP admissions were based on WHZ alone [27]. Secondary analysis of CMAM data from other areas of South Sudan found that 47% and 34% of children with SAM, respectively, were identified based on WHZ or MUAC criteria alone with only 19% of children meeting both criteria [28]. Both studies discussed the possibility of increasing the MUAC threshold for OTP admissions (to increase sensitivity for both mortality and low WHZ admissions), and also noted implications in terms of increased caseload. Recent evidence reviews describe the strong body of evidence for MUAC (with edema) as the sole criterion for identification and discharge, with both noting further research is needed, particularly in relation to optimizing context-specific admission thresholds that maximize mortality reduction and sustained recovery within the local context, and in consideration of resource limitations and high levels of unmet needs [6,7]. Findings from this study align with evidence from South Sudan and suggest that if MUAC-only admission/exit criteria are adopted, a thorough review of evidence from South Sudan is needed to inform the identification of optimal MUAC thresholds.

In contrast to many simplified protocols that have been trialed, the COVID-19 protocol in South Sudan did not include reduced dosing over the course of recovery, and instead use fixed dosing over the full treatment course, meaning that lower-weight children received additional RUTF early in OTP treatment, which may have been beneficial. In this study, simplified dosing under the modified protocol included two RUTF sachets daily in OTP which differs from most modified dosing strategies where dosage is reduced over the course of recovery [6]. Under the standard OTP protocol where weight-based dosing is used, smaller amounts of RUTFs are provided early in treatment, with dosage gradually increasing as the child’s weight rises. Various dosing regimens have been explored with the aim of improving recovery rates and reducing non-response, and existing evidence suggests that recovery rates using modified dosage (reduced over time) are non-inferior to recovery rates using weight-based dosing [6,7,29]. One potential explanation for the observed higher recovery rates and shorter LoS is the larger dosage amount under the modified protocol early in recovery (the difference in dosage is greatest for lower-weight and younger children). One concern with providing a set amount of RUTF irrespective of weight is that older/larger children may be disadvantaged and have worse outcomes because they receive less RUF than with weight-based dosing. 

Limitations: There are several important limitations to consider when interpreting the results of this study that hinder the ability to draw strong conclusions. There were only seven sites in total, which requires caution when interpreting results given potentially strong clustering. The facilities are in a single county and operated by one organization and cannot be broadly generalized. Due to the inability to randomize, observed differences may be attributable to variations in treatment facilities and/or the populations in their catchment areas. Because sites under the standard protocol were participating in a separate ongoing study, additional resources and attention may have been provided to these sites leading to variations in program implementation. Included covariates may not be exhaustive of potential confounders in the study, meaning these factors are unaccounted for in models. When excluding WHZ admissions in the standard protocol, differences persist in TSFP model results for LoS and recovery. In theory, MAM treatment under the two protocols should be the same for MUAC-admitted children (one RUSF sachet per day with bi-weekly follow-up); this suggests that outcomes may be attributable to unobserved differences between sites and not to the two protocols. Finally, it was not possible to follow children after program exit to assess if relapse rates differed between the protocols.

## 5. Conclusions

The presented study is the first to evaluate the performance of modified CMAM protocols in the context of the COVID-19 pandemic prospectively, allowing for adjustment for child and household characteristics. In this study of CMAM treatment during COVID-19 in seven health facilities in South Sudan, children treated under the modified protocol had higher recovery rates and LoS compared to those treated under the standard protocol. Point estimates from models adjusting for differences at the site, individual and household levels suggest a similar direction and magnitude of effect; however, adjusted estimates were not significant. Age was not associated with the odds of recovery under either protocol. It is important to note that populations eligible for treatment under the modified protocol differ from the standard protocol due to the use of MUAC-only admission criteria, compared to MUAC and WHZ under the standard protocol. Differences in the population eligible for treatment may also have contributed to the observed differences in program outcomes. 

These results align with two other recent studies that show improved recovery rates for acute malnutrition during COVID-19 in South Sudan, which is a strong evidence base that suggests within a pandemic context, simplified treatment protocols may be beneficial. Any modification to CMAM programming should carefully consider MUAC thresholds for admissions/discharge to ensure the inclusion of wasted children. Given the contextual differences between the COVID-19 pandemic and the present, further examination of how simplified protocols perform is necessary to improve nutrition-program outcomes in South Sudan.

## Figures and Tables

**Table 1:** Standard and COVID-19-adapted treatment protocols.

	Standard Treatment Protocol	COVID-19 Modified Protocol
Admission Criteria	MUAC AND/OR WHZ AND/OR bilateral pedal edema	MUAC AND/OR bilateral pedal edema
	OTP: MUAC < 115 mm, WHZ < −3 TSFP: MUAC 115–124 mm, −3 ≤ WHZ <−2	OTP: MUAC < 115 mm TSFP: MUAC 115–124 mm
Simplified Ration Dosing	OTP: Weight-based RUTF dosing TSFP: One RUSF sachet daily	OTP: Two RUTF sachets per day TSFP: One RUSF sachet daily
	OTP: Weekly distribution TSFP: Bi-weekly distribution	OTP: Weekly distribution TSFP: Bi-weekly distribution
Discharge Criteria	Meets MUAC or WHZ criteria (same criteria as used for admission) for two consecutive visits	Meets MUAC criteria for two consecutive visits
	OTP: MUAC ≥ 115 mm OR WHZ ≥ −3 TSFP: MUAC ≥ 12.5 cm OR WHZ ≥ −2	OTP: MUAC ≥ 115 mm TSFP: MUAC ≥ 125 mm

MUAC: mid-upper arm circumference; OTP: outpatient therapeutic program; RUSF: ready-to-use supplementary food; RUTF: ready-to-use therapeutic food; TSFP: therapeutic supplementary feeding program; WHZ: weight/height z-score.

**Table 2:** Characteristics of children and their households at study enrollment.

	All Children			Enrollment at OTP			Enrollment at TSFP		
	Standard CMAM	COVID Modified	*p*-Value	Standard CMAM	COVID Modified	*p*-Value	Standard CMAM	COVID Modified	*p*-Value
	*n* = 515	*n* = 594		*n* = 226	*n* = 239		*n* = 289	*n* = 355	
Child characteristics									
Female	57.3%	54.9%	0.42	53.1%	54.8%	0.71	60.5%	54.9%	0.15
Age in months, mean ± SD	18.0 ± 9.7	20.5 ± 11.2	<0.001	18.3 ± 10.3	19.7 ± 11.3	0.18	17.7 ± 9.3	21.0 ± 11.0	<0.001
<24 months	76.3%	65.5%	<0.001	74.8%	66.7%	0.26	77.5%	64.8%	<0.001
≥24 months	23.7%	34.5%		25.2%	33.3%		22.5%	35.2%	
Currently breastfed	68.3%	63.0%	0.03	68.1%	65.7%	0.57	68.5%	61.1%	0.05
Caregiver characteristics									
Caregiver is female	98.5%	100%	0.002	99.1%	100%	0.15	97.9%	100%	0.006
Caregiver age									
≤18 years	1.9%	2.4%	0.008	1.8%	2.9%	0.02	2.1%	2.0%	0.20
19–37 years	74.6%	71.0%		72.1%	69.0%		76.5%	72.4%	
38–58 years	13.6%	10.3%		14.6%	8.4%		12.8%	11.6%	
Age unknown	9.9%	16.3%		11.5%	19.7%		8.7%	14.1%	
Caregiver married	95.7%	86.5%	<0.001	96.9%	83.7%	<0.001	94.8%	88.5%	0.004
Caregiver has no formal education	77.7%	81.0%	0.17	80.1%	84.5%	0.21	75.8%	78.6%	0.40
Household characteristics									
Female head of household	71.8%	75.3%	0.20	67.7%	78.7%	0.008	75.1%	73.0%	0.54
Household size, mean ± SD	7.7 ± 4.6	9.8 ± 10.3	<0.001	8.1 ± 6.5	10.3 ± 12.0	0.01	7.4 ± 2.3	9.4 ± 8.9	<0.001
Currently displaced	5.8%	3.2%	0.03	6.2%	0.8%	0.002	5.6%	4.8%	0.66
Received assistance in past 3 months	7.4%	4.9%	0.08	7.5%	3.8%	0.08	7.3%	5.6%	0.40
Household hunger score, mean ± SD	2.2 ± 1.1	2.7 ± 0.9	<0.001	2.1 ± 1.1	2.7 ± 0.9	<0.001	2.3 ± 1.1	2.7 ± 0.9	<0.001
Little to no hunger	25.1%	11.4%	<0.001	30.4%	12.9%	<0.001	20.9%	10.4%	0.001
Moderate hunger	72.6%	85.8%		67.0%	85.8%		77.0%	85.8%	
Severe hunger	2.4%	2.8%		2.7%	1.3%		2.1%	3.8%	
Meals consumed the previous day, mean ± SD	1.5 ± 0.6	1.6 ± 0.6	0.05	1.6 ± 0.5	1.7 ± 0.6	0.08	1.5 ± 0.6	1.6 ± 0.6	0.17
Distance to a treatment facility (km), mean ± SD	5.7 ± 4.8	5.1 ± 4.3	0.01	5.6 ± 5.0	5.0 ± 4.3	0.20	5.9 ± 4.6	5.1 ± 4.2	0.03

CMAM: Community management of acute malnutrition; km: kilometers; OTP: outpatient therapeutic program; SD: standard deviation; and TSFP: therapeutic supplementary feeding program.

**Table 3:** Nutritional status and treatment outcome by treatment and protocol.

	OTP Treatment			TSFP Treatment		
	Standard CMAM	COVID Modified	*p*-Value	Standard CMAM	COVID Modified	*p*-Value
	*n* = 226	*n* = 239		*n* = 429	*n* = 471	
Admission anthropometry						
MUAC, mean ± SD	11.5 ± 0.6	11.2 ± 0.3	<0.001	12.1 ± 0.3	12.1 ± 0.2	0.13
MUAC < 11.5 cm	58.9%	100%		0.0%	0.0%	
11.5 cm ≤ MUAC < 12.5 cm	39.4%	0.0%		90.0%	100%	
MUAC ≥ 12.5 cm	1.8%	0.0%		10.0%	0.0%	
WHZ, mean ± SD	(−3.2) ± 0.8			(−2.1) ± 0.7		
WHZ < −3	67.7%			7.0%		
−3 ≤ WHZ < −2	24.3%			51.4%		
WHZ ≥ −2	8.0%			41.6%		
Discharge anthropometry						
MUAC, mean ± SD	12.0 ± 0.5	11.8 ± 0.2	<0.001	12.6 ± 0.6	12.6 ± 0.5	0.82
MUAC < 11.5 cm	8.9%	6.3%		7.2%	8.1%	
11.5 cm ≤ MUAC < 12.5 cm	72.6%	92.9%		17.5%	12.1%	
MUAC ≥ 12.5 cm	18.6%	0.8%		75.3%	79.8%	
Total MUAC gain (cm)	0.5 ± 0.4	0.6 ± 0.3	0.02	0.5 ± 0.5	0.5 ± 0.5	0.55
Average MUAC gain per day (mm/day)	0.16 ± 0.2	0.2 ± 0.2	<0.001	0.07 ± 0.1	0.1 ± 0.2	0.002
Time in treatment						
Length of stay (days) Mean ± SD	42.8 ± 21.7	38.3 ± 24.1	0.03	61.9 ± 21.9	54.0 ± 21.7	<0.001
Median (IQR) ^†^	35 (29)	28 (32)	<0.001	58 (35)	55 (33)	<0.001
Time to recovery (days) Mean ± SD	40.2 ± 18.5	37.0 ± 23.0	0.12	61.3 ± 19.0	51.2 ± 19.7	<0.001
Median (IQR) ^†^	35 (22)	28 (28)	<0.001	58 (27.5)	50 (29.5)	<0.001
Time to recovery (days) excluding LoS > 90 Mean ± SD	39.0 ± 16.8	34.1 ± 19.0	0.006	57.7 ± 15.9	50.4 ± 18.9	<0.001
Median (IQR) ^†^	35 (22)	28 (22)	<0.001	58 (27)	49 (30)	<0.001
Discharge outcomes *						
Recovered	87.2%	93.3%	0.03	72.7%	79.8%	0.01
Default	0.0%	0.0%		8.9%	7.6%	
Traditional default	0.0%	0.0%		1.6%	1.1%	
Default due to stock-out	0.0%	0.0%		7.2%	6.6%	
Non-responsive	11.5%	5.0%		4.2%	1.9%	
Transferred						
Transfer to OTP	0.0%	0.0%		8.4%	7.2%	
Transfer to a new facility	0.4%	0.4%		2.6%	2.1%	
Transfer to inpatient care	0.4%	0.8%		0.2%	0.0%	
Death	0.0%	0.0%		0.0%	0.2%	
Improper/Early discharge	0.4%	0.4%		3.0%	1.1%	

cm: centimeters; CMAM: community management of acute malnutrition; IQR: interquartile range; LoS: length of stay; mm: millimeters; MUAC: mid-upper arm circumference; OTP: outpatient therapeutic program; SD: standard deviation; TSFP: therapeutic supplementary feeding program; and WHZ: weight/height z-score. * Based on a 120-day maximum length of stay, nine children were excluded from analysis due to a length of stay exceeding 120 days. ^†^ *p*-values obtained using the two-sample Wilcoxon rank–sum tests.

**Table 4:** Unadjusted and adjusted differences in length of stay and odds of recovery.

	OTP Treatment				TSFP Treatment			
	Standard	Modified			Standard	Modified		
Unadjusted difference	Mean LoS (days)		Difference		Mean LoS (days)		Difference	
COVID-modified protocol *	42.9	38.3	−4.8	(−20.8, 9.1)	61.8	54.0	−6.92	(−21.6, 9.0)
Adjusted difference **								
COVID-modified protocol			−9.95	(−25.3, 4.7)			−7.75	(−21.7, 6.8)
Age at admission (months)			0.7	(−2.3, 3.8)			−0.2	(−2.2, 1.8)
Female, sex			−0.1	(−4.1, 3.9)			−1.7	(−4.4, 1.1)
MUAC at admission (mm)			−1.1	(−1.5, −0.6)			−1.3	(−1.8, −0.8)
Unadjusted odds	Recovery Rate (%)		Odds of Recovery		Recovery Rate (%)		Odds of Recovery	
COVID-modified protocol *	86.7	93.3	2.09	(0.77, 5.60)	72.7	79.8	1.37	(0.47, 3.98)
Adjusted odds **								
COVID-modified protocol			2.63	(0.70, 9.09)			1.80	(0.42, 7.24)
Age at admission (months)			1.08	(0.55, 2.13)			1.09	(0.76, 1.55)
Female, sex			1.20	(0.72, 2.12)			0.77	(0.59, 1.00)
MUAC at admission (mm)			1.16	(1.08, 1.25)			1.28	(1.19, 1.38)

LoS: length of stay; mm: millimeters; MUAC: mid-upper arm circumference; OTP: outpatient therapeutic program; and TSFP: therapeutic supplementary feeding program. * Standard CMAM protocol is used as a comparison group, which includes random effect for the facility of admission. ** Adjusted for facility of admission as random effect and child age, child sex, MUAC at admission, household size, household hunger score, current displacement status, caregiver age, previous episode of acute malnutrition, currently breastfed, number of years in community, number of children in household, education of caregiver (some primary or more), marital status of caregiver, number of meals in previous day, treatment site distance, and assistance received in past three months prior to enrollment as fixed effect.

## Data Availability

The dataset supporting the conclusions of this article is available in the Humanitarian Data Exchange, and can be accessed at https://data.humdata.org/dataset/ssd-aah-final-dataset (accessed on 1 September 2023).

## Appendix A

**Table A1:** Unadjusted and adjusted differences in length of stay and odds of recovery: MUAC admissions only.

	OTP Treatment				TSFP Treatment			
	Standard	Modified			Standard	Modified		
Unadjusted difference	Mean LoS (days)		Difference		Mean LoS (days)		Difference	
COVID-modified protocol *	45.5	38.3	−6.88	(−21.8, 8.0)	60.5	54.0	−6.52	(−22.1, 9.1)
Adjusted difference **								
COVID-modified protocol			−6.44	(−21.1, 7.6)			−5.08	(−19.4, 8.5)
Age at admission (months)			0.3	(−3.3, 3.8)			−0.1	(−2.2, 2.0)
Female, sex			1.3	(−3.5, 6.1)			−1.0	(−3.8, 1.8)
MUAC at admission (mm)			−1.9	(−2.6, −1.1)			−2.2	(−2.8,−1.6)
Unadjusted odds	Recovery Rate (%)		Odds of Recovery		Recovery Rate (%)		Odds of Recovery	
COVID-modified protocol *	83.3	93.3	2.67	(0.99, 6.82)	72.9	79.8	1.57	(0.60, 4.20)
Adjusted odds **								
COVID-modified protocol			2.07	(0.39, 9.57)			1.55	(0.35, 6.71)
Age at admission (months)			0.89	(0.39, 2.06)			1.19	(0.79, 1.79)
Female, sex			1.44	(0.77, 2.78)			0.72	(0.53, 0.97)
MUAC at admission (mm)			1.28	(1.15, 1.44)			1.40	(1.28, 1.54)

LoS: length of stay; mm: millimeters; MUAC: mid-upper arm circumference; OTP: outpatient therapeutic program; and TSFP: therapeutic supplementary feeding program. * Standard CMAM protocol is used as comparison group; this includes random effect for the facility of admission. ** Adjusted for facility of admission are random effect and child age, child sex, MUAC at admission, household size, household hunger score, current displacement status, caregiver age, previous episode of acute malnutrition, currently breastfed, number of years in community, number of children in household, education of caregiver (some primary or more), marital status of caregiver, number of meals in previous day, treatment site distance, and assistance received in past three months prior to enrollment as fixed effect.
